# “I Just Find It Easier to Let Go of Anger”: Reflections on the Ways in Which Yoga Influences How Young People Manage Their Emotions

**DOI:** 10.3389/fpsyg.2021.729588

**Published:** 2021-11-22

**Authors:** Ingunn Hagen, Solbjørg Skjelstad, Usha Sidana Nayar

**Affiliations:** ^1^Department of Psychology, Norwegian University of Science and Technology (NTNU), Trondheim, Norway; ^2^Stabilisati Counseling Service, Psychological Counseling, Yoga and Meditation Guidance, Levanger, Norway; ^3^Former Professor in Psychology, Tata Institute of Social Sciences (TISS), Mumbai, India

**Keywords:** yoga research, yoga in school, young people, mental health and well-being, managing emotions, emotional regulation, emotional processing

## Abstract

In this article we discuss how young people experienced a school-based yoga intervention. We pay particular attention to how yoga provides a space for young people to deal with their emotions. We base our discussion on qualitative data from young people in Norway who participated in the European research project “Hippocampus: Promoting Mental Health and Wellbeing among Young People through Yoga.” The qualitative results are based on experiences described by these young people in individual semi-structured interviews and in diaries or logs. Our data include nine interviews performed in the spring of 2019 with young people of Norwegian and refugee background in their late teens and early twenties. There were also 133 logs noted by the students exposed to the yoga intervention. In the qualitative interviews, young people talk about yoga and emotional management, improved sleep habits, and regulation. They also report improved ability to regulate and cope with stress. Yoga seemed especially beneficial for refugee trauma. In this article, we have chosen to focus on the utterances of young people about emotions, as those were quite dominant in our data, especially in the interview material. We have identified instances of emotional regulation, but also of emotional processes and changes of emotions, all of which were related to these young students practicing yoga. The impact of yoga on emotions illustrates the potential of yoga to improve the well-being and mental health of young people.

## Introduction

Young people face many internal and external changes which can make them hyper-emotional, and the period of adolescence brings significant challenges in dealing with these emotions. In this article, we will address how yoga seems to improve the processing and regulation of emotions of people based on qualitative interviews and logs linked to yoga interventions in school settings. Our data were collected as part of the five country European research project, ‘‘Hippocampus: Promoting Mental Health and Wellbeing among Young People through Yoga.’’ The Hippocampus project involved Norway, Belgium, Italy, Spain, and the United Kingdom and was financed by the Erasmus+ program, European Union (EU)^1^. Our article focuses on the qualitative results from the Norwegian Hippocampus project. We find focusing on yoga and emotions important as successful emotional regulation is closely linked to well-being (Menezes et al., 2015a). Emotional health is also important for learning and for positive social interactions (Khalsa and Butzer, 2016).

The intention of the Hippocampus project was to give young people tools to cope with the challenges and stressors in their everyday lives. Eight-week yoga courses were provided as interventions, mainly in school settings. The ancient techniques of yoga can be described as a ‘‘technology of wellbeing^2^.’’ Here, yoga contains both physical and mental poses and practices, like asanas, meditation, and pranayama (breath and energy control), along with guidelines for conduct toward others and self-study (Yamas and Niyamas)^3^. These guidelines are part of Yoga Philosophy and may help young people to develop Life Mastery and life skills^4^. The Hippocampus project aimed to investigate how practicing yoga impacted the well-being, stress, and sleep of young people. In the project, we were also interested in how disadvantaged young people experienced yoga when it was incorporated as part of their school classes. In the project, disadvantage was identified as problems related to mental health, physical health, psycho-social, and/or socioeconomic.

### Emotions of Young People

In this part of the article, we will address what characterizes the adolescence period of life in terms of emotions. Generally, the teenage years are characterized by emotional turmoil as there is a greater activation of the emotional part of the brain (the limbic system) during adolescence. In a recent article, Guyer et al. (2016: 74) suggest that adolescents are highly emotional and thus are often “hijacked by their emotions.” These authors describe how the emotional life of adolescents is complex both from the inside and outside. From the inside, young people experience both hormonal changes and “fine tuning of the neural networks that both produce and manage their emotions” (2016: 75). From the outside, young people often experience dramatic changes in their social relationships (parents, peers, romantic relationships, etc.) along with the increasing demands of school and society.

Due to this high level of emotionality, the adolescent years are characterized by an “increase in psychopathological levels of dysfunctional affect” (Guyer et al., 2016: 75). To learn to manage one’s emotional reactions is an important developmental task for young people for their socio-cognitive development. How they develop in these areas will impact their emotional decision-making and self-regulation skills (see Martinus, 2018). Guyer et al. (2016) describe adolescence as a period of hyper-emotionality, which often includes increased risk taking and reward seeking. Such a period of heightened emotionality, where some experiences can be like emotional roller coasters, will often impact the well-being and mental health of young people. Adolescence is not only a period of increased emotionality, but also affect-related psychopathology (see Guyer et al., 2016). Thus, how young people relate to their emotions is important for their overall well-being.

In which ways do young people and others relate to or manage their emotions? We will describe two forms of managing emotions here, which will also be discussed in the context of our data. *Emotional regulation* is perhaps the most well-known concept and refers to the way individuals manage an experience and express their emotions (cf. Peluso and Freund, 2018). In other words, emotional regulation describes the ability to both adjust emotional reactions to socially accepted expressions and, at the same time, allow for flexibility in the responses of an individual. Related to practicing yoga, emotional regulation implies that individuals may develop the ability to override emotions in favor of more adaptive responses (see Gailliot et al., 2010; Hagen et al., 2018).

*Emotional processing*, on the other hand, “refers to how individuals experience, organize, make meaning of, and resolve emotional episodes” (Peluso and Freund, 2018: 2). Thus, emotional processing refers to a more raw, direct experiencing of the internal landscape of emotions, witnessing them occur, fade away, and sometimes shift into other emotions or even into neutrality. Thus, emotional processing presupposes a certain level of acceptance toward the immediate emotional reaction to the situational stimuli. Here, acceptance refers to having a non-judgmental attitude toward one’s own immediate emotional response and feelings. In therapies that focus on emotions, such as Emotion Focused Therapy (EFT), processing is often discussed with regard to management of difficult emotions (see Greenberg, 2015). There is an increasing understanding within research and practice of psychotherapy of the fact that inattention or avoidance toward emotions is associated with mental health difficulties (Stiegler, 2018).

### Well-Being and Mental Health of Young People

As indicated, in the Hippocampus project we were interested in how young people experienced yoga provided for them in a school setting while paying particular attention to those in situations of disadvantage. Thus, we need to discuss the situation of young people in Norway, both in terms of emotional and everyday life challenges, along with their well-being and mental health. As a way of approaching disadvantage, we will address mental health challenges of ethnic Norwegian youth and those with immigrant and refugee background.

In terms of well-being, it is worth noting that Norway was ranked number one by the World Happiness Report in 2017^5^. In fact, Norway has several times that have been ranked among the top ten countries of the world in terms of the happiness of its population^6^. Contributing factors include Norway as a wealthy and rather egalitarian country with a functioning welfare society. However, such national rankings of happiness are merely pre-conditions for the experiences of happiness and well-being of the population.

Many people in Norway are also in situations of disadvantage when it comes to well-being and mental health, and, generally, young people are at a more vulnerable stage of life than the adult population. As in many countries, young people in Norway live in a media saturated environment, and are beset by sleep problems, issues with body image, and expectations of high performance, all of which may impact their well-being and mental health. In fact, the young generation in Norway has been labeled Generation Performance (Madsen, 2018) or even Generation Perfect (Hegna et al., 2013). As these terms imply young people may internalize expectations from their school, parents, society, and, especially, those ideals conveyed through social media to the extent that they feel under constant pressure (Nayar et al., 2012).

For some young people, these internalized expectations and pressures can lead to dissatisfaction with life and mental health problems. When discussing mental health, it may be useful to distinguish between three categories or levels of severity. One is psychological disorders which correspond to diagnostic criteria, such as depression, anxiety, ADHD, etc. The second is psychological or emotional problems which are not diagnosed but are still hampering normal functioning at school and in life. Notably, about two-thirds of young people suffer from stress, emotional problems, and reduced functioning (Helland and Mathiesen, 2009). The third category is mental well-being, where young people feel happy, content, and that they have a meaningful life. We propose that young people in the first two above-mentioned categories (having a diagnosis or experiencing psychological/emotional problems) are in situations of disadvantage.

Recent national reports on young people in Norway suggest that most young people are content and happy with their lives and experience well-being and good mental health (e.g., Bakken, 2019)^7^. However, there are also increasing mental health problems, rising drug consumption and violence, increasing experiences of loneliness, and less optimism about the future, and these are contextualized by more screen time and use of social media. Moreover, fewer young people experience well-being at school with an increasing number of students who regard it as boring, or dreading having to attend it. Girls, in particular, seem to experience school-related stress, psycho-somatic health problems, worrying, and having depressive symptoms. The longitudinal Norwegian Trøndelag Health study (HUNT-study) confirms that mental health problems and stress are increasing, and that girls especially are experiencing increasing levels of anxiety and depression (Sund et al., 2019). Boys, on the other hand, seem to be more affected by challenges like ADHD^8^.

What about the well-being and mental health of immigrant and refugee youth, who are also participants of our study? Immigration to Norway has increased in recent years, with the share of population currently around 18%^9^. Most immigrants are generally satisfied with their lives in Norway, though, as a group, they tend to have greater disadvantages, including psycho-social challenges, mental health problems, and socio-economic problems. However, immigrants in Norway are a diverse group, varying from people coming to work or study to refugees escaping conflicts and war. Immigrant youth and refugees will have many of the same mental health problems as other Norwegian young people, along with some that are specific to their life experiences as refugees who have often faced life threatening situations and traumatic experiences. Of interest for our study is the fact that half of unaccompanied refugee minors have symptoms of post-traumatic stress disorder (PTSD) when they arrive in Norway (Grøholt et al., 2018).

### Can Yoga in School Settings Improve the Mental Health and Well-Being of Young People?

What does research say about yoga offered to young people in school settings? In a review article, Khalsa and Butzer (2016: 45) suggest that “yoga in the school setting is a viable and potentially efficacious strategy for improving child and adolescent health and therefore worthy of continued research.” A recent review article by Miller et al. (2020: 1) confirms that yoga is a promising intervention for children and young people. These authors also find “that yoga has positive effects on a range of outcomes in psychological/behavioral, cognitive, and physiological/physical functioning” (p. 13). Indeed, practicing yoga seems to improve mental health and well-being in and beyond school settings (Hagen and Nayar, 2014; Nanthakumar, 2018). Researchers also find that yoga facilitates learning among young people (Serwacki and Cook-Cottone, 2012; Frank et al., 2014; Morgan, 2014; Butzer et al., 2015; Childress and Harper, 2015; Khalsa and Butzer, 2016; Balkrishna et al., 2019). While schools are emphasized as ideal settings for promoting healthy lifestyle skills from an early age (Khalsa and Butzer, 2016), there is less clarity about the styles of yoga, advice on frequency, and how it works. Still, scholars do seem to agree about the value of teaching yoga as a holistic system of practices, including asanas or physical postures, pranayama or breathing exercises, relaxation techniques, and meditation or mindfulness practices (op.cit. p. 46).

We now turn to yoga and its impact on emotions, the central theme in this article. Miller et al. (2020) were to find that practicing yoga increase the ability to regulate emotional, cognitive, and somatic impulses and experiences. Similarly, in a review article on yoga and emotion regulation, Menezes et al. draw to the conclusion: “emerging evidence suggests that yoga may help foster healthier psychological responses, indicating its potential as an emotion regulating strategy” (2015a: 82). Based on an overview and comparison of yoga-based inventions in the United States, Butzer et al. (2015), conclude that yoga improves socio-emotional skills, along with performance and behavior in the classroom. Mental health and well-being of young people include the ability to self-regulate emotionally, mentally, behaviorally, and to develop healthy relationships with peers and teachers. Other researchers find that yoga in schools led students to have improvements of mood and self-regulation skills for emotions and stress, and, thus, to have better resilience (Khalsa et al., 2012; Khalsa, 2013; see also Noggle et al., 2012). As we have seen, better mood and being able to deal with emotions and cope with stress are important for the development of life mastery skills and well-being of adolescents.

One of the early qualitative studies of yoga in school (Conboy et al., 2013) report the following psychological benefits for students: “many cited stress reduction; many used yoga to manage negative emotions; and some propagated more optimism” (p. 171). Butzer et al. (2015) praise qualitative research for providing an in-depth perspective on the impact of yoga interventions in schools. Yoga can provide positive and pedagogical support in school and “increase mindfulness, emotional regulation, and positive behaviors of school students” (Accardo, 2017). In a study of mindful yoga, Bergen-Cico et al. (2015) conclude that “systematic, sustained training, and practice of yoga and meditation integrated into academic classes can cultivate self-regulation among emerging adolescents” (p. 11). However, in a study by Butzer et al. (2017), the students had more mixed feelings about yoga, but also reported that yoga facilitated self-regulation and academic performance. A study by Novaes et al. (2020) conclude that yogic breathing or pranayama impacted emotions in the form of reduced anxiety and increased positive emotions. These authors find the parts of the brain like the amygdala, anterior cingulate, anterial insula, and pre-frontal cortex activated and connected in processing emotions after yoga. They also find that awareness and attention play a role in this.

Related to the mental health and well-being of young people, yoga can both be health promoting and disease preventive (Velásquez et al., 2015). Yoga fosters awareness and acceptance and is a method to teach young people to be more aware of their own breath, body, and mind, and to be more self-conscious (Flak and de Coulon, 2011; Butzer et al., 2015). Research suggests that yoga may decrease anxiety among children and adolescents (Weaver and Darragh, 2015), and enhance emotion regulation skills (Daly et al., 2015; Khalsa, 2015). Practicing yoga increase the awareness of young people to their body, mind, emotions, and reaction patterns, as was demonstrated in a study of yoga to promote the mental health and well-being of teenagers and young adults (see Hagen et al., 2018). In their review article on yoga and emotional regulation, Menezes et al. (2015a) suggest that psychological improvements “occur through the interplay between the activities that yoga incorporates, such as controlled breathing, mindful postures, relaxation, and, in some cases, formal meditation, which may lead to the development or enhancement of emotion regulation skills” (2015a: 94). These authors found that attention regulation (through the pre-frontal cortex), acceptance, regulation of autonomic activity (reduced anxiety and arousal), and endocrine responses underlie the relationship between yoga and emotion regulation. Increased awareness about themselves and their emotions can be valuable for young people to increase their resilience and to promote their mental health and well-being. The way yoga impacts how young people manage their emotions beyond emotional regulation is what we want to examine and discuss further in the current article.

### The Hippocampus Project

The Hippocampus project, which the present study was a part of, consisted of both intervention and research parts. The aim of the Hippocampus intervention, as described in our project proposal, was defined as ‘‘to develop and implement an appropriate yoga-based approach to bring these benefits to young people in situations of disadvantage.’’ Five European countries, Belgium, Italy, Norway, Spain, and the United Kingdom were involved^10^. The idea was to reach so-called disadvantaged youth by reaching all young people, mainly in school contexts. An integral approach was sought by also involving teachers. The goal was to improve the well-being of young people, including disadvantaged youth, by offering them weekly yoga sessions for 8 weeks. In total, the yoga intervention program reached over 750 young people and teachers.

The 8-week Hippocampus yoga program introduced young people (and teachers) to new asanas every week, with each yoga session having a different purpose, namely, in week 1, nourishing was in focus; in week 2, the intention was building focus; in week 3, the aim was to build trust; in week 4, the intention was that young people listened to themselves with compassion; in week 5, the focus was on promoting optimism; in week 6, the purpose was to promote Metta or loving kindness; in week 7, radical self-care was in focus; and in week 8, the goal was to motivate the young people to continue with their self-practice of yoga. Each week was supposed to build on the previous week so that the values and poses were repeated, providing the young people with a yoga “starter toolkit” for developing life skills. The yoga teachers were also advised to promote the eight limbs of yoga emphasized by the well-known codifier of yoga, Maharishi Patanjali in the *Yoga Sutras* (see Hagen, 2018)^11^. The limbs pranayama, asana, and meditation were all integrated in the classes.

This 8-week Hippocampus yoga program was developed by one of the project partners, United Kingdom-based Charlotta Martinus^12^. As preparation, Martinus ran a 5-day training course for five certified yoga teachers, one from each of the countries involved. In Norway, seven more yoga teachers were recruited to fit with the schedules of the four schools participating. These seven yoga teachers were taught to deliver the 8-week Hippocampus yoga program course locally. The yoga teacher course was accompanied by a manual describing the yoga program for young people along with the yoga program for staff and/or teachers (see text footnote 2). There was also a parallel development of an instructional app *Yuva Yoga*, based on the Hippocampus yoga intervention. The app was intended to be a resource to stimulate further practice of yoga, but it was completed too late to be part of the intervention. The app content is nevertheless available in the five project languages (see García-Holgado et al., 2019).

## Materials and Methods

The research part of the Hippocampus project was to investigate the potential impact of practicing yoga for both young people and their teachers. The Hippocampus project combined quantitative and qualitative data in a mixed method approach. We briefly describe the quantitative part of the study as a background for our focus on the qualitative part, where there was a chance to go more into depth through qualitative interviews (the interview guide is in the Appendix). The qualitative data also included logs where the students were encouraged to write about their experiences with yoga in their own words.

For the Hippocampus survey, there was a pre and post-intervention design. Both young people and their teachers received a questionnaire before and after the 8-week yoga intervention. The questionnaire was provided to all young people who were students in the yoga classes and, also, to involved teachers, who participated in their own yoga classes after school^13^. The qualitative data consisted of a variety of approaches: semi-structured qualitative individual interviews (with disadvantaged youth), group interview (teachers), diaries or logs (young people, only in Norway), and reflection notes (yoga teachers).

The Hippocampus Project had an integral design where the purpose was to integrate yoga in the culture of each participating school or organization by making participation in an 8-week yoga course available to teenagers and their teachers. The yoga interventions in Norway reached 201 pupils and 49 teachers in four different schools in Trondheim. For this article, we focus on the qualitative data from young people and will not go into the group interview with teachers. The data with students include nine interviews performed in the spring of 2019 with young people of Norwegian and refugee background in their late teens and early twenties. There were also 133 logs noted by the students exposed to the yoga intervention.

The project was designed to reach the so-called disadvantaged youth in their natural environment. Being disadvantaged, as we mentioned earlier, had been defined in the Hippocampus project as a young person between 13 and 25 years old, in a situation characterized by a challenge in one or more of the four aspects: mental health, physical health, psycho-social, and socioeconomic factors. Mental health, psycho-social, and socioeconomic factors are the three aspects our interviewees are challenged by. As mentioned above, our data include nine qualitative interviews with disadvantages young people. Three ethnic Norwegian youth experienced mental health conditions (anxiety and ADHD), while one of the Norwegian interviewees was bicultural and described as “difficult in class.” In addition to these four, there were five refugee interviewees with psycho-social and socioeconomic challenges, as well as different degrees of trauma, coming as they did from war zones such as Afghanistan, Kurdistan, Syria and Somalia.

The 8-week yoga course was first introduced at a High School (HS1) and an International School (IS) during the fall of 2018. HS1 is a “university high school,” frequently cooperating with the largest university of Norway, NTNU, for projects, educational programs, and teacher training. IS is an International Baccalaureate school, with children that have one or both parents originating from other countries. In 2019, we also recruited teenagers and teachers at a secondary school (SS). In order to secure participants for the interviewees characterized as disadvantages youth, we recruited students with some mental health challenges. We also recruited participants in young “adult learning programs” for migrants and refugees, who attended two classes at another High School (HS2) and one introductory class at HS1. Thus, we recruited migrants and refugees from two different school program levels: one group of newly arrived refugees of high school age following the primary school curriculum at HS2, and one group participating in an introductory school program at HS1 which was focusing on a class high school curriculum and more skilled at speaking and writing in Norwegian.

The nine individual qualitative interviews were conducted with young people who had experienced some form of disadvantaged circumstance in their lives. For the recruitment for the interviews, we followed the guidelines from the project agreement (i.e., meeting at least one of the disadvantaged criteria mentioned above). We started with an open invitation to participate in interviews in the information letter and consent form. We also requested assistance for recruitment to the qualitative interviews from yoga teachers and coordinating teachers, explicating that we were interested in young people in situations of disadvantage.

Our recruited sample of interviewees is a result of these combined recruitment effort and the distribution of project information, along with students volunteering to participate. Our sample for the qualitative interviews consisted of nine students with certain disadvantages, as mentioned earlier, such as having mental health challenges and/or refugee background. Four of the interviewees were students with an ethnic Norwegian background, namely, a Norwegian high school girl who volunteered, introducing herself as diagnosed with ADHD, a girl diagnosed with anxiety, a boy diagnosed with ADHD in secondary school (SS) were recruited by their informed class teacher, and a bicultural boy perceived as difficult in class was recruited by his teacher at IS.

Five of the students in our interviews had a background as immigrants, mainly refugees seeking asylum in Norway. Newly arrived refugees also qualify as psychosocially disadvantaged, both by limitations of Norwegian language and culture. Many of them also carry traumatic memories of war, have experienced loss of close family, or has even lived in Norway as an underaged refugee without any family. The interviews with the three refugees least skilled in the Norwegian language were conducted with a mother language assistant who were staff hired at HS2 to facilitate communication and learning in the languages Dari, Arabic, and Somali. The extracts from the interviews are given in verbatim to better illustrate the experience of the teenagers. Translations made by mother language assistants (MLAs) may be found after that CMSAs abbreviation.

All the qualitative interviews were performed by Solbjørg Skjelstad, and they took place in the various school settings of each student for a duration of approximately 30 min. We also requested that all the students who participated in the yoga course to write logs where they were asked to share their experiences related to practicing yoga in school in diary form to write more freely. 133 of the young students chose to do so. These logs also form part of our qualitative data, supplementing the data from the qualitative interviews. The instructions for the logs were given by the different yoga teachers, and they vary somewhat accordingly. However, the overarching idea of the different instructions was to encourage the students to articulate their experiences with yoga in their own words, thus, hopefully giving us insight into how yoga influenced their mental and emotional health.

The analysis of the Norwegian qualitative data based on the interviews was mainly thematic, based on national data, while also cooperating with the teams of the other countries at the initial stage. The joint process of analyzing the qualitative interviews was conducted by manual coding in the five national languages which the teams decided was necessary for retaining possible semantic nuances. Then, following a coding scheme, identifying actualized themes according to well-being at school and with family and friends, stress, and sleep was done based on the interview guide^14^. For the Norwegian coding of the qualitative interviews, we have applied a three-layer coding process: descriptive, conceptual, and theoretical (Kvale, 1994). We will describe these themes below and pay particular attention to how the informants experienced the impact of yoga on how they dealt with their emotions^15^.

## Results

### Background Results

One of the most important themes in the Norwegian qualitative interview material was, indeed, the emphasis the young interviewees put on how yoga impacted the way that they dealt with their emotions, something that we will discuss below. But first we will briefly present the quantitative data as a background for our discussion based on the qualitative data. Generally, the quantitative results for all five countries of the Hippocampus project after the yoga intervention showed an improvement in well-being, reduction in perceived stress, and fewer sleep problems for both young people and staff compared to the baseline. These results were statistically significant, but the effect sizes for these changes were moderate (see Hippocampusproject.eu), and the Norwegian effect sizes were smaller than for the five-country sample.

While 413 young people in the overall five country Hippocampus survey responded to the baseline dataset (T1), 339 young people responded to the follow-up (T2). Due to various factors of attrition, the matched dataset became 260. The corresponding numbers in the Norwegian survey with young people were 133 (T1), 87 (T2), and 40 for the matched dataset. In the analysis of the Norwegian data, no statistically significant differences between T1 and T2 could be found, partly as a result of the attrition between T1 and T2. Still, there was a tendency after the 8-week yoga course in Norway toward improved well-being and sleep, along with decreased perceived stress.

Thus, we find it important to explore further how yoga was experienced by young people through the qualitative data where they express their yoga experiences in their own words. The Norwegian qualitative data with young people consists of nine individual semi-structured interviews and logs or diaries from 133 informants. Below, we will present the qualitative interviewees from the Norwegian part of the Hippocampus project by first giving a short presentation of the two groups of teenagers participating in the interview, and then paying attention to the results based on our thematic analysis of the qualitative interviews and logs.

### Presentation of the Interviewees^16^

Firstly, we will provide a short description of the interviewees, namely, four Norwegian born participants and five migrants/refugees. Next, we will present data from these nine individual interviews and another layer of data collected by asking the students participating to write logs or diaries about their experience with yoga.

Fay was a Norwegian high school girl (HS1) who volunteered as an interviewee, disclosing to her yoga teacher that she has been diagnosed with ADHD. She reported that she had had good experience with previously using meditation but expressed that yoga deepens the tools she was already familiar with: *“It’s the inner peace, since I’ve definitely gotten stronger after doing yoga (.) yes, Inner strength, mentally and yes, just mental strength and that I am calmer.”*

Emily was a pupil at the secondary school (SS) who reported experiencing anxiety while she still seemed to be popular and well-functioning. She revealed that she had experience with meditation and breathing exercises she learned from apps along with input from coaches and therapists to cope with panic attacks. Emily expressed that in order for her to really enjoy the benefits of the relaxation part at the end of a yoga class, she needed the tempo of the yoga class to be paced well for her, particularly, to be slow enough:

*The minutes when I’m there it seems to be helpful, but it helps the most when I’m calm and not super stressed. The breathing exercises and the mindfulness that we do, that works kinda fine, but the exercises [asanas] just make me, if we do them to hasty then it only makes me more stressed.*

David, a boy with ADHD also attended secondary school. Both he and Emily were asked by their teacher if they would like to volunteer as interview persons for the qualitative interviews in the project. David reported that he found yoga to be both helpful and soothing: *“I manage to not be stressed if it was a test for example. Just sitting relaxed. Because then you can think of something else*… *just when you just do like this with your body (sits still in meditation pose, eyes closed).”*

Another boy from the international secondary school, Cole, was recruited by his teacher who also participated in the project as yoga teacher. His disadvantage was described as “being difficult in class.” Otherwise, he seemed to be popular and thriving, maybe with a need to show off. Cole shared some interesting perspectives on awareness during his experience with Yoga Nidra, a relaxation exercise inviting calm alertness: *“You are aware of what is happening, but you don’t pay attention to what is happening. When we do Yoga Nidra, it feels like you’re falling backward when you are not moving at all. It’s not like bad weird, it’s like(.) cozy and relaxing.”*

Ayla and Barbara were two girls from Syria attending an “introductory class” for migrants in an ordinary Norwegian high school. These two girls had some skills and knowledge of the Norwegian language. Ayla expressed: *“I am very lucky to have learnt yoga. I can forget about the war and choose the positive things and thoughts*.” Barbara shared:

*(laughs) erm, for example. school too, when we for example shall do a presentation or something, then will go well, you can do that, I do like this (breathes in, lifts arms and taps her fingers) and it becomes better. Erm, yes and what I said about controlling myself and not be stressed, and be calm and yes, just to have less stress.*

Javid from Afghanistan, Hugir from Kurdistan, and Idil, a girl from Somalia, all attended the same adult learning program for migrants with no basic education. More concretely, they were participating in primary school educational programs within a high school environment. Javid was an unaccompanied refugee minor, seemingly struggling with memories from war and worries about family members still living in Afghanistan. He stated that he had found yoga to be helpful: *“When I have a headache, then you try yoga, and it gets very good and cozy.”* Javid expresses that he has grown very fond of yoga: *“100% recommends yoga. Yoga is the 100% best, recommends everyone does yoga.”* Kurdish Hugir was a boy who liked to work out and who had integrated yoga into his exercises at the gym: *“Yes, I’ve learned something I can use. (.) I just think about yoga, I’m at the gym, I do yoga. It’s at the gym with two friends. I say you can do like this (laughs) this is yoga.”* Idil was the Somali girl with a big smile but was still reserved. She expressed that she had found yoga to be mostly helpful for sleep and relaxation, and she loved the sensation of the focused breathing in yoga. She described: *“Breathe slowly with the belly. Just the belly that flows.* Interviewer inquired into what she experienced: “*And that relaxes your body?,”* and she described further: *“Yes, my fingers, my feet, all of them*.”

### Qualitative Research Results

In this part, we will discuss how yoga seemed to impact the way young people dealt with their emotions, primarily based on the Norwegian qualitative interviews, and on the logs. As a background, we will briefly mention the other themes identified in the qualitative interviews with young people in the five European countries taking part in the Hippocampus project, “Promoting Mental Health and Wellbeing among Young People through Yoga.” The key common themes included (1) *Calm, (2) Emotional dimension, (3) Focus and Concentration*, and *(4) Awareness.* The themes in the international collaborative coding coincided with the Norwegian manual coding in NTNU. In the present analyses, we also included NVivo 12 for quality assurance. The “Emotional dimension” was much more emphasized in the Norwegian qualitative interviews, something we found interesting and wanted to explore further. The theme “emotional dimension” generally refers to all incidents of young people discussing how their emotions were affected and regulated by the practice of yoga.

There are four overarching themes that stand out as the most dominant and interesting in the Norwegian qualitative interview material: (1) ‘‘*Yoga as emotional management*,’’ (2) ‘‘*Yoga as recognition of need for relaxation*,’’ (3) *‘‘Yoga as regulation of sleep habits,’’ and* (4) *‘‘Yoga as beneficial for refugee trauma.’’^17^* The last-mentioned theme is drawn from the data of two groups of migrants participating in our interviews. In the current article, the focus is on yoga and its potential impact on emotional management, which will be elaborated below.

### Yoga as Emotional Management—Emotional Regulation and Processing

Our nine individual interviews had a clear theme of “*Emotional management*” which contained the subthemes “*Emotional Processing*” and “*Emotional Regulation.*” We find these subthemes intriguing and decided to explore further as emotional health and emotional education research keeps growing and are important fields of knowledge to develop for ensuring relational competence and psychological health (Butzer et al., 2015). We will briefly repeat the notion of *emotional management* and the distinction between emotional processing and emotional regulation, which we presented in the introduction, before we present citation from the interviewees and the log material.

Emotional management is a theme containing several subthemes concerning how the teens use yoga to handle their emotions. We have chosen to highlight and distinguish between two different approaches on how the interviewees discuss of directing attention inward toward how they feel and react, labeling the subthemes *emotional regulation* and *emotional processing.* Although both emotional regulation and emotional processing involve emotional change, we have chosen *emotional changes* as a general label for the instances where the informants describe changes in emotional states in the log material. From our qualitative data, there are indications of yoga being a potential space for allowing awareness of the emotional inner landscape of an individual, and being a tool of emotional self-regulation enabling appropriate, socially acceptable expressions and ways of being. We will investigate these nuances in more detail below.

### Yoga and Emotional Regulation

The conscious choice of regulating emotions by means of the well-being technology of yoga, as it emerges in our data, seems to be a sort of reorganization of the inner emotional landscape through focusing on breath or cognitively embracing the value of keeping calm. We see several instances of this in our interview material.

David showed us how he regulated his waves of stress and anger through elements of what he had learned during the yoga intervention:

*That if you are stressed and angry, just stop and breath. Then you think about something else. Then you think about the breathing. It (the anger) kind of disappears. It’s just to stop and kinda*… *it’s a bit difficult to explain*… *it’s like you throw it (the anger) away. You kinda just stop and think a little about*… *And then not think at all. That you just breathe. Find a new solution or because that kinda, I have anger problems because I have ADHD*.

What he described here is that he has discovered that focusing on his breath like he had learned in the 8-week yoga course influences how he dealt with his anger. He also referred to the fact that when all focus was on the breath, his thoughts calmed down along with his anger. Pranayama, breathing exercises and synchronizing focus, movement, and breathing were central skills learned in the yoga course, and he seemed to have discovered a way to become aware of other ways of perceiving a situation “find a new solution” than acting out his anger. If redirecting his attention to breath helps him manage his anger as he described, it could become a valuable life skill.

David found tools for personal agency in yoga that directly address his anger: *“I don’t have to get so angry anymore. I can choose to channel the energy into sports and other activities.”* David also found it useful when regulating nerves before a school test: *“I manage to not be stressed if it was a test for example. Just sitting relaxed. Because then you can think of something else*… *just when you just do like this with your body [sits still in meditation pose, breathes deeper, eyes closed].”* He referred to how he made use of relaxation techniques from the yoga classes to calm his nerves, finding a relaxed sitting position while breathing deeply (a classic end of yoga classes).

Cole shared how he found it easier to regulate his need to act out on irritations occurring in daily life: *“Maybe I’ve become. I don’t get so easily angered. For example, if my sister is very irritating, so maybe I get like (shakes his head) no, that won’t affect me.”* Here, he responded to a question asking whether doing yoga had influenced how he interacted with his family. Similarly, Emily tells us about a shift in how she speaks with her mother by reminding herself to keep calm:

*I’ve become better at being able to talk calmly, not raising my voice. If mum raises her voice to me, I can feel I get irritated, but then I just talk calmly, and try to just, just, get like, like, that she must tell me what it is so that I can understand, and manage to kind of talk calmly if we are having a discussion.*

Cole and Emily seemed to have discovered the strength and value of keeping calm, that they had a choice of whether to react or not, and that they could be able to choose how they respond. As mentioned, meditation and self-conduct are central parts of yoga, and are touched upon in the 8-week Hippocampus program used as an intervention. Apart from the relational value of choosing to be calm in meetings with conflict and anger, these statements can be interpreted as deliberate regulations of emotional activation. They could also be regarded as an initial improvement of emotional health in their ability to recognize the difference between being emotionally triggered, acting out on their emotions, and finding that they are able to rather act calmly, which can be preferable.

Ayla and Emily shared the awareness of the usefulness of keeping calm and regulating the anger just by being reminded of the equanimity and calm they were introduced to in yoga classes: *“Not to be angry at something. To talk calmly, yes, because that is the problem. You have to become calm. That when I’m angry at something, calming, calming, like I do in yoga. Yes. It’s not good to talk bad to others. Then they don’t feel good inside.”* It seemed like yoga had an experiential effect on the young people teaching them to handle the emotions popping up in new ways through the effect of being exposed to the system, behavior, and ambiance of the eight yoga classes.

Fay, who was diagnosed with ADHD, explained that she had found that yoga helped her to create a space between herself and the experience of strong emotions:

*Before I had a lot of mood swings, where I could go from extremely hyperactive and couldn’t sleep for 3 days to being totally down all the time and feel very sick. But by not thinking about anything at all in a way, manage to keep your mind just calm, I managed to have my feelings more under control in a way.*

She responded to a question about whether yoga had influenced her sleep quality, and here we see quite a remarkable shift into being responsible for herself in a new way. Particularly, by focusing on the value of keeping calm and seemingly experiencing a form of control over her emotional life. Generally, the routine of yoga has the potential to relieve excess energy, sharpen focus, and withdraw attention from anything going on externally, shifting instead to what is going on internally. Thus, there may be space to perceive different impacts on emotion regardless of whether these comes from outside or inside.

For Fay, who as mentioned was diagnosed with ADHD breathing exercises helped her to distinguish triggers and focus on what she can control:

*And when I’m in public, I’ve had a bit of problems with anxiety before, and then I use the breathing exercises and, in a way focus more on myself. The thought that, like, that it’s real all around me, when I like I am at a full buss and you kind’a get anxious but like it’s only other persons with feelings too, and they can be just as anxious.*

Rather than just focusing on her own anxiousness, Fay seemed to regulate herself by acknowledging that others might have similar feelings. This was a way of normalizing and relating herself to a common human experience rather than relating to her feelings with shame or fear of being different. We will now focus on the other nuance of dealing with or managing emotions, where these shifts happen without the interviewees deliberately willing or deciding to change their emotions and moods.

### Yoga a Potential Space for Emotional Processing?

Yoga can be a practice of action and reflection. Yoga can also be an invitation to connect with how you respond to life and how you develop as a person. It is particularly a form of open dialog with the whole of your being, with body, mind, and breath. Moreover, yoga provides training in observing the state of mind and emotions of an individual, and contains the mentioned guidelines for living well—a set of ethics named Yama and Niyama (see Hagen, 2018; Martinus, 2018). The Hippocampus Manual included these guidelines, encouraging initial attitudes of acceptance, self-care, and focusing on the present moment. Processing or witnessing emotions has the potentially transformational effect of realizing that one is not necessarily identified as being the emotions, but rather that the emotions pop up as a response to the present circumstance and then changes into something else when exposed to other circumstances, activities, and relations (Siegel, 2010). We will demonstrate what we interpret as the witnessing element of processing emotions below.

A transition into a more pleasant emotional state induced by practicing yoga without deliberately regulating emotions was described by Ayla saying: *“I don’t need to think about the negative things when I do yoga. So, yoga made me feel comfortable inside.”* She was aware of carrying negative memories and emotions, but still not focusing in on them. One could perhaps understand this quote as an avoidance of negative thoughts and, thereby, unpleasant emotions, or rather, with a pursuit of pleasant emotions despite many negative aspects of her life situation. But it is equally possible to interpret this quote as Ayla letting everything be as it is. Particularly, being enabled to tap into the here and now and the movement, focus, and breath during a yoga class and experiencing greater comfort as a result. The comment also suggested a less comfortable state of body and mind before entering the yoga class, and since Ayla is a refugee from Syria, the comment bears even more weight. She speaks of sadness because of loss and war in the interview and finds that yoga gives her an option to let emotions be recognized and transformed during the 45 min of yoga interventions each week: *“One day I was very sad and so tired and sleepy, but I had yoga, and after that I felt, ‘ah, how nice I am happy and feel comfortable’.”* We read this as an effortless change in well-being and emotions that she allows to happen during the practice of yoga, and that the opportunity of being led through a series of movements and coordinated breathing and focus gives her access to moments of happiness and well-being. She does not seem to be suppressing her sadness and tiredness but experience a transition into a more positive emotional state for a while. This coordination of movement, focus, and breath is the space for processing we find interesting.

Ayla also reflected on how her emotional transformation “on the mat” let her shift her mind set, perhaps only temporarily, but still in a way that enabled her to reconnect to a latent positive mindset: *“It was good to do yoga when I was sad and stuff, so I don’t have to have negative thoughts. Yes, just look at the positive side.”* She also shared how a group of friends within the yoga class could lighten up and seemingly feel an aftermath of effects: *“Yes, we were in a good mood, we became happy. Yes, and laughs of a lot of things.”*

These reports from the refugees stood out because we know from the interviews that they were grounded in memories of war and family back in their home country. Nevertheless, our Norwegian interviews also spoke about the perceived shift in emotional state, as David, the boy diagnosed with ADHD, described:

*“Yes, I have fewer negative thoughts, and am more positive and efficient and such” Feels like after I have done it, I kind of look more forward to, am excited about going home to a friend afterward or be at home, play, be outside. Yes, I get more kinda positive. mm, focus on something positive.*

Again, we find that the quote potentially pointed to an observation of being in an improved mood. Specifically, that negative thoughts were not in focus but rather anticipating and looking forward to potential positive experiences and interactions. He did not speak about trying to think positively or avoiding unpleasant emotions but suggested that it happens naturally after yoga. It is our interpretation that he refers to a form of witnessing his own thinking and mood when he starts class. He does not regulate but was rather able to access excitement and appreciation of what happens during his day. This is an example of how yoga can provide an emotional transition through synchronized movement, focus, and breath.

Furthermore, David had recognized how a yoga class can make him shift mood, and how he experienced some sort of reset similar to waking up to a new day fully rested: *“I felt a bit sleepy, in a good way. It’s like, it becomes a bit like that in the eyes*… *Feels like, because it feels almost like I wake up, because if you have a bad day, and then you go to sleep and then you wake up and then everything is fine again. It feels a bit like that.”* It seems like he experienced the shift of mood lasting for the rest of the day and even improving his engagement with schoolwork: *“It makes you happy. Like when you feel happy and look forward to something. What happens often is that I look forward to going and do stuff and I do pretty much better at school as well. Em*…, *and I feel positive.”*

Fay revealed during the interview that most of her friends were outside the school environment and that she did not feel very connected to her classmates but she had noticed a shift in the group of teenagers doing yoga together: *“But I see they are more open in a way. They’re not*… *there was a while when they weren’t that negative, and I appreciated that. Always when we were done, we were happy and just talked to each other and were very open.”* There seemed to be a new openness toward each other and, perhaps, a form of kindness was made available when the operating mode was that of parasympathetic nervous system as characterized by relaxation response rather than a sympathetic nervous system response of alertness. Such a change in group dynamics was not a deliberate regulation of emotions, but possibly an effect of changes in moods and being relaxed by sharing the activity of yoga. Fay had also experienced this shift within herself as being set free from bothersome emotions, not by intending to move away from them, but by the sheer activity of yoga asanas followed by the *Savasana (resting) pose* and relaxation. *“I just got very calm (by doing relaxation after yoga). Many started to fall asleep, you just got that inner calm. And that no matter if the day had been very bad, you kind of finish with what feelings had built up and you’ve in a way been set free.”* It seemed that yoga created a group dynamic where kindness, support and feeling safe was something both traumatized refugees and Norwegian teenagers struggling with psychological problems and disorders experienced as an effect.

### The Logs: Yoga Experience and Changes in Emotions

The logs were intended as a form of diary for reflecting on how yoga influenced the well-being of young people. The students were encouraged to write in their yoga logs before or after the 45 min Yoga class and to store the logs at school. Generally, the participants were requested to write about their experiences before, during, and after yoga class along with how they felt about doing yoga. As in the interviews, the logs referred to various versions of managing emotions and experiences of a change in mood from negatively charged into positively charged emotions. We will stick with description in this section and leave the writings of the participants to speak for themselves as further instances of the emotional changes reported in the interviews. The format of personal log writing seemed to invite the participants to reflect on the felt experience of yoga and gave the quotes a more immediate character: *‘‘Now I soon walk out the door and feel more rested and in a better mood.’’* Another student wrote: *‘‘I think my body feels lighter, and I feel my sad thoughts have gotten av bit smaller.’’* Some of the young people were quite specific when referring the helpful aspects of the yoga class: *‘‘I think Metta was lovely, and the exercise made me happy.’’^18^* Others observed that moods can change or be modified by doing yoga: *“Today I was pretty irritated and angry, Yoga helped a little.”*

One student described how the yoga class helped her feel better after the loss of a family member and experiencing various difficulties amongst her friends:

*Today I feel better than yesterday because I was thinking of a family member who was only minutes away and who died this weekend. It has been very heavy for me. Heavy time. A lot of friends have heart ache and depression. Friends are cutting themselves. What can I do about that kinda? All the stress around me right now. I want yoga every week.*

Even though the student did not explicitly say yoga made the heavy times and surroundings easier, we interpret «*I want yoga every week*» as a statement that suggest finding some sort of support, relief, or solace that can make stress and heaviness easier to manage, thereby causing participants to feel better after yoga class.

Another student found yoga to be helpful when worrying about a parent: *“Yesterday my dad ended up in the hospital, I’ve been worried, tired, scared and sad the last day, but yoga made me relax and forget about the worrying.”* It seemed that being in a yoga class allows for relaxation and soothing rather than avoidance of difficult emotions, while also being a space where troubles could wait for a while.

In addition, some of the students reflected on how yoga helps them with difficult feelings: *“Today was also very calm and relaxing. And it has helped me calm down a bit. I need calm. I think this has helped me with stress and anxiety*. *And it is good to get an hour during the school day to relax. It has also helped me when I get depressed.”* As also mentioned above in the interview data, it is the neural regulation in which yoga stimulates that can relieve emotional states such as heightened levels of anxiety and sadness. Among the more well-integrated refugees at the introductory class at CH high school, there was still a distinctly different way to express how yoga was helpful—where the need to let go of negativity and feel comfortable and focused was shared: *“All negative thoughts are gone after yoga,”* Another person expressed: *“It was difficult to manage to focus that much, but I could do it for a bit.”* Doing yoga seems to change feelings to the better: *“It’s comfortable. Thinking better. It made me happy. I feel a bit better after yoga. I want to sleep.”* Moreover, practicing yoga also make young people aware of the need to sleep: *“I am sick. Sleepy, tired, want to sleep, afraid, stressed. After yoga I felt comfortable, happy, want to sleep. I think yoga is a good way to get comfortable.”* As these log comments indicated, yoga seemed to allow young people to feel better about themselves and their lives even if only for a moment of the day. They are reminded that it is possible, and that they can acquire tools and life skills to help them feel better.

## Discussion

In this article, the main contribution is exploring how practicing yoga through a school intervention made a difference in how young people managed their emotions. Managing emotions was a central theme in our qualitative data, which was collected as part of the Norwegian Hippocampus project. Based on the Norwegian Hippocampus survey data, we found minor but not significant impacts of the yoga intervention on the wellbeing, experienced stress, and sleep of young participants. It was a limitation in our Norwegian part of the study that the quantitative data was not strong enough to allow for our intended triangulation. When we further explored the qualitative data, however, we identified interesting results regarding how yoga seemed to impact the way our young interviewees and informants dealt with their emotions. In fact, responses about managing emotions were one of the central themes in the qualitative interviews we performed with young people in Norway. Statements about emotions were also central in the log material where the young people wrote about their experiences related to yoga^19^.

Due to the fact that we have two sources of qualitative data, interviews and diaries/logs allowed us to get both interview data with chosen individuals in situations of disadvantage and more spontaneous logs from a larger number of the young people who were part of our Hippocampus project. The focus of informants on emotions is not so surprising as the teen years are often characterized by emotional turmoil and, as mentioned in the introduction, young people can be “hijacked by their emotions” (Guyer et al., 2016). How young people manage their emotions is important for their wellbeing and mental health. Thus, we found it important to examine how disadvantaged and other young people implement elements from what they learnt during an 8-week yoga intervention in school into daily encounters with emotionally challenging situations and memories. Managing emotions was a central theme, and the distinction between processing and regulating emotions indicated in our data is a potentially fruitful area for further research related to the well-being technology of yoga. The notion of processing emotions may contribute to a deeper understanding of how yoga can influence emotional health. In the logs it was especially the changes from negative to more positive emotions that our informants found worth mentioning. We interpret these changes in emotions as related to the space for relaxation during parts of the yoga sessions, as less arousal reduces emotional reactivity (Menezes et al., 2015a).

In line with suggestions from other researchers in the field, the young people in our study were offered a holistic version of yoga, including yoga philosophy. The Hippocampus manual applied by the yoga teachers for teaching the 8-week yoga course to the young people in this study also included elements from Patanjali’s *Yoga Sutra*, namely Yama and Niyama, which are the first two of the so-called eight limbs of yoga (Hagen, 2018). Thus, the Hippocampus project exemplified how even an introductory course can convey yoga as a useful way of life through introducing the attitudes and values provided in the ethics of yoga^20^. Yama and Niyama describing a set of restrictions or social behavior and a set of personal behavior or routines for cultivating a harmonious inner life, respectively, can perhaps serve to further illustrate the difference between emotional regulation and emotional processing. Regulation and Yama points toward a restriction and correction of behavior to adapt to social interaction, while processing and Niyama point toward an inner process of personal guidelines for a harmonious and natural lifestyle. This is particularly the case in applying elements of yoga such as various tools for focusing in on breath, known as Pranayama, and Pratyahara (control of your senses) as ways to disengage the mind by controlling reactions to external disturbances (Hagen, 2018). In the following section, we will discuss how we identified nuances of emotional management, and why we find it useful to make the distinction between emotional regulation and processing.

In the qualitative results of the Norwegian Hippocampus study, young people described how yoga enabled them to manage their anger, reduce anxiety, experience less depression, feel calmer, and happier, etc. Yogic breathing was often mentioned as contributing positively in terms of being with and dealing with emotions. When focusing on how these young people described their management of emotions, we paid attention to the distinction between regulating and processing emotions. Regulating emotions implies more deliberately choosing to manage feelings and emotions according to social norms and expectations in order to more efficiently operate and navigate in relations to others and the world. Processing emotions on the other hand, has more of a witnessing quality of registering what reactions and emotions occur within along with observing how the popping up of emotions naturally moves into new sensations and emotions during the activity of focusing on the practice of yoga where movement, breath, and focus are synchronized. A central aspect of the witnessing quality of emotions for enabling a processing of emotions includes the allowance to accept difficult along with positive and pleasant emotions. The notion of processing emotions is more in line with how therapeutic traditions of client-centered and emotion-focused practices approach a lived experience that involves difficult emotions rather than a more goal oriented and cognitive regulation of the emotions into a wanted state of mind or behavior (Pascual-Leone, 2018). During our research, we found indications of emotional experiences other than deliberate regulation or avoidance of unpleasant emotions and have thus chosen to shed a light on this nuance.

In the research literature on yoga in school and yoga related to the mental health and wellbeing of young people, several authors discuss how practicing yoga seems to increase the ability of young people to regulate their emotions (Khalsa et al., 2012; Noggle et al., 2012; Khalsa, 2013, 2015; Daly et al., 2015; Menezes et al., 2015a,b; Hagen et al., 2018; Miller et al., 2020). However, to our knowledge, none of the yoga researchers pay attention to what we call emotional processing. Our data illustrate that yoga may provide space for emotional processing, where the allowance of being with what is and being attentive to, yet not identified with the experienced emotion, seems to be accentuated during the practice. In such a state within the frame of yoga practice, following one’s emotions can stimulate activation of other emotions in such a way that the emotional information and affect is received as input, and the problematic emotional state starts to be disentangled instead of shutting down. We understand emotional processing as a more inward sequence of emotions, an internal management of sometimes difficult emotions, and allowing for both the difficult and the positive emotions to be simultaneously present. Regulating emotions, on the other hand, is an outward regulation of expressing socially accepted and appropriate emotional reactions according to situational interactions and relations. Processing emotions requires an acceptance and witnessing mode. It seems that yoga is a potential space or sanctuary for experiencing and feeling what you indeed feel. In modern and traditional yoga classes, there can be certain “politically correct” expectations that doing yoga should result in becoming “happy and peaceful,” which might inspire participants to act a certain way or trigger behavior that is socially desirability. But accepting the present moment as it is, being with what is truly present can lead to less reactivity to negative emotional stimuli as well as reducing emotional suppression and rumination (Menezes et al., 2015a). This creates the conditions for the yoga classroom to be a space for emotional witnessing and processing, along with discovering how the yoga practice provides tools and life skills to handle unwelcome and unpleasant emotions.

Practicing yoga implies taking life and emotions ‘‘to the mat,’’ to digest life through consciously moving, breathing, directing mental focus, and becoming aware of the state of mind and emotions. In contrast to the deliberate intention to regulate the intensity or quality of emotions described above under emotional regulation, we identify in the data a witnessing quality of being able to be with the unpleasant emotion and then experiencing a possible shift into a more neutral, accepting emotional state or releasing into a positive, more content state of mind, rather than an avoidance of or suppression of difficult emotions. We see an allowance of pleasant and relaxing states of mind and the activity of the autonomous nervous system to come to the fore^21^. When Ayla, the Syrian girl, went to the mat sad, tired, and sleepy and afterward expressed with some surprise “*but I had yoga, and after that I felt, ‘ah, how nice I am happy and feel comfortable’*,” she experienced a transition happening during yoga, and not a deliberate attempt to regulate herself into feeling happy or avoiding feeling sad. Perhaps even befriending the difficult emotion, or changing the relationship with anxiety, sadness, or anger, and finding value in the potentially important information about self, identity and situation reside in being honest with the self and developing clarity on what one feels about the present moment. The focus here being on experiencing and registering what is taking place and witnessing it changing, letting one emotion move, and transform into another or simply be as it is without any willed effort to make it happen. For instance, when Fay found herself somehow freed of accumulated emotions: “*And that no matter if the day had been very bad, when you kind of finish with what feelings had built up and you’ve in a way been set free.”* While emotional processing is mainly a therapeutic concept, emotions are allowed to inform and affect the individual (Greenberg, 2015). The vital aspect of emotional processing in a therapeutic frame is *attention* toward the *emotional activation* of an individual (Stiegler, 2018). This resembles yoga as space for emotional processing, where being with what is and being attentive to, yet not identified with the experienced emotion, seems to be an accentuated. In such a state within the frame of yoga practice, allowing the emotions of an individual simply to be can stimulate further activation of emotions in such a way that the emotional information is received as input and the eventual problematic emotional state potentially starts disentangling instead of being shut down.

Emotions are important because they motivate and give directions to our actions, they inform us about our human relations and about the fulfillment of our needs (Greenberg, 2015; Binder, 2018). Thus, to be able to be aware of and process emotions is important for young people, as it allows them to take important decisions in their lives and to deeply connect with other people. The ability to be aware of one’s own emotions and those of others may be regarded as a particular skill, sometimes referred to as emotional intelligence (cf. Goleman, 2005). To be mindful of one’s own body, like in yoga, can be a way to be aware of and embrace one’s own emotions without judgment. According to Binder (2018), such awareness can make us realize that emotions are transient. They will come and go.

Researchers have also found that positive emotions have a beneficial role for cognitive processing, while negative emotions harm coping mechanisms and create stress (Narasimhan et al., 2011). Young people have been found to conflate stress with negative emotions, and it is beneficial that yoga interventions contribute to improved impulse control and emotional regulation, and thus fewer conflicts with family and peers (Dariotis et al., 2016a). For young people, it can be valuable to develop improved emotion regulation skills, to de-escalate negative emotions and create more calmness and wellbeing (Dariotis et al., 2016b). The ability to allow emotions to inform and affect oneself, whether the emotion is difficult or positive, due to creating an attitude of acceptance and an environment encouraging “being with what is” in a yoga class, can potentially be a form of processing emotions to prevent problematic emotional states or unprocessed emotions.

Practicing yoga enables one to become aware of the body, emotions, and mind of an individual, and also to develop an “awareness of awareness” (cf. Gitananda, 1981). Thus, when practicing yoga, one often develops skills to be aware of emotions and thoughts as they arise. Based on the Hippocampus yoga intervention, the young people participating in the project seemed to go through the following aspects of emotional awareness: develop awareness of emotions, feel able to process or witness emotions, and when needed, regulate emotions. In our study, we found that young people became more aware of their emotions, something which is important as a life skill in itself. Awareness of emotions often allowed the teenagers to evaluate their emotions, something which facilitated the further management of their emotions. In this way practicing yoga could empower young people to regulate their emotions in beneficial ways, along with getting to know themselves better and acknowledging vulnerability and strengths in an environment or space where experiencing a spectrum of emotions are accepted and thus normalized. Practicing yoga also leads to better balance between the parasympathetic and sympathetic part of the autonomic nervous system, and often less activation of the limbic system. Thus, feeling more calm, less activated, and more in “rest and digest mode” may make the experience of being young less of an emotional rollercoaster. As was demonstrated in our data, with their guards down, the youngsters felt more positive and also noticed that others were more open and approachable. This, again, facilitated more of a psychological sense of community, something that would likely be appreciated by peer-focused young people.

Especially promising in the present study are the reports from teenagers implementing elements from what they learn during the 8-week yoga intervention in school into daily encounters with emotionally challenging situations, and from refugees finding useful ways to deal with memories of war. The explicit use of different techniques of pranayama and focus on breath and practicing breath-based meditation and mindfulness are relatively available, and these simple tools for emotional, spiritual, and cognitive support can benefit young people today. The impact of yoga on the emotions of people has received increasing attention in recent years. Other studies report that practicing yoga has reduced negative emotions, like depression, anxiety, anger, and also that practicing yoga can increase the experiences of people of positive emotions, like joy and well-being. For example, Narasimhan et al. (2011), and Felver et al. (2015) find that yoga led to significantly decreases in anger, anxiety, depression, and fatigue, along with improved well-being among adolescents in school. We also see increased joy and well-being and reduction in anger, anxiety, and sadness in the results presented above from the Norwegian qualitative data, based on interviews with and log data from young people. There are also numerous studies that focus on how practicing yoga can lead to an improvement in the ability of people to regulate their emotions. That is, to modulate the intensity and expression of emotions to make them more socially acceptable, and thus lay the groundwork for healthy relationships, social interaction, and learning. As mentioned above, several authors find evidence that yoga has a potential for increasing the ability of young people to regulate their emotions and, thus, create healthier psychological responses (e.g., Menezes et al., 2015a). Similarly, the informants in our study report of increased ability to regulate emotions, such as feeling and expressing frustration and anger, something that seems to increase their well-being and self-esteem.

There were some limitations of the study that this article was based on, being part of the European research project “Hippocampus: Promoting Mental Health and Wellbeing among Young People through Yoga.” Due high attrition for the Norwegian quantitative data, we did not find it meaningful to include those data in the current article, and thus implement the mixed-method design that we had aimed for. Still, while the quantitative data did not result in statistically significant findings, they indicated tendencies toward increased well-being, less stress, and improvement on sleep habits after the yoga intervention. For the qualitative interviews, we think that it could have been useful to have a clearer definition of disadvantaged young people in the Hippocampus project, something that would have influenced our recruitment of informants for the qualitative interviews. Being participants in an international research project both give privileged access to colleagues, knowledge, methodologies, and discussions, while also, to some extent, limiting the degrees of freedom in research design. Still, the interview guides were the starting point for semi-structured interviews where our informants provided insights into how they experienced a yoga intervention in their school setting. As we have demonstrated in the current article through the qualitative interviews, the young people in situations of disadvantage shared with us how yoga impacted the way they managed, processed, and regulated their emotions. Also in the logs, many of the young people chose to focus on how yoga influenced the way they dealt with emotions and of changes in emotions. When interviewing the young refugees who did not know Norwegian or English very well, we had to rely on mother tongue assistants due to limited time and economic resources. In hindsight, we think that the interviews would have benefited from having more professional interpreters.

In this article, we have addressed how yoga impacts how our young informants dealt with their emotions by discussing instances where they regulate and process emotions, and where emotions change. Focus on emotions is valuable, because “how one feels and reacts to and expresses emotions can have both short and long-term effects on physical and mental health” (Menezes et al., 2015a). This is explained by such mechanisms as reappraisal, attention regulation, self-monitoring, self-awareness, and regulation of the autonomic nervous system. As yoga reduces negative emotions like anxiety, anger, and depression, this is likely to result in less conflict and stress in the lives of young people. We think that it is also important for young people to be able to accept difficult emotions and allow them to be acknowledged and validated. When negative emotions are acknowledged and witnessed, they often dissolve or transform, and the process allows the individual to learn about their own boundaries, preferences, and needs. Processing emotions in this way allows for honesty with oneself and may contribute to a healthier development. Thus, yoga seems to be a useful well-being technology and practice that schools should welcome, as it could increase life skills for their students, such as concentration, memory, relational skills, and decision making, all of which are influenced by emotions. Emotional wellbeing is important for learning in life and in school. As discussed earlier, research supports such a claim, even though more research is needed to understand how and why yoga should be offered to young people in their school. However, we encourage researchers to further explore the role of yoga in the management of emotions, both in terms of emotional processing and regulating emotions. The role of yogic breathing (pranayama) also needs to be explored further, as part of a holistic view of yoga, and especially the role of yoga in the relationship between being with emotions, regulating emotions and how this relates to change.

### Notes of Gratitude

This article is dedicated to our dear friend and colleague Professor Usha Sidana Nayar, who passed away in 2021. She was a co-worker in the Hippocampus project and has also been a mentor and source of inspiration for many years. Furthermore, we would like to express our gratitude to all the students, teachers and yoga teachers who took part in the Norwegian part of the Hippocampus project. In addition, we are grateful to our partners in Belgium, Great Britain, Italy, and Spain, as well as the students, teachers, and yoga teachers from these countries. Participants from two countries deserve special thanks; Nick Kearny for initiating the project together with Charlotta Martinus, who also developed the 8-week yoga course and the manual. Thanks also to the Spanish team for securing funding for the Hippocampus project and for developing the Yuva Yoga App. Finally, thanks to NTNU and the Department of Psychology, NTNU for providing extra funding and a supportive work environment during the Hippocampus project period and beyond.

## Data Availability Statement

The raw data supporting the conclusions of this article will be made available by the authors, without undue reservation.

## Ethics Statement

The studies involving human participants were reviewed and approved by NSD—Norwegian Centre for Research Data. Written informed consent to participate in this study was provided by the participants’ legal guardian/next of kin.

## Author Contributions

IH was contributed to the design and planning of the research project and wrote the draft of the article, including the data analysis provided by SS. SS took on the main responsibility for data collection and did the data analysis of the qualitative data, with input from IH and UN and wrote a summary report of the qualitative data analysis. SS and UN contributed to the revision of the article drafts, with both theoretical concepts and organizational aspects. IH and SS have finalized the article draft. All authors contributed to the article.

## Conflict of Interest

The authors declare that the research was conducted in the absence of any commercial or financial relationships that could be construed as a potential conflict of interest.

## Publisher’s Note

All claims expressed in this article are solely those of the authors and do not necessarily represent those of their affiliated organizations, or those of the publisher, the editors and the reviewers. Any product that may be evaluated in this article, or claim that may be made by its manufacturer, is not guaranteed or endorsed by the publisher.

## Footnotes

1 see Hippocampusproject.eu

2 This term was coined by Nick Kearny in the Hippocampus project proposal. It is also used in the Hippocampus manual (see Hippocampusproject.eu).

3 The Yamas and Niyamas are part of the eight limbs of yoga, a central element in Patanjali’s *Yoga Sutras*, the most quoted source of Indian Yoga Philosophy (see Hagen, 2018). Yamas refer to five social ethics: *ahimsa/*non-violence, *satya/*truthfulness, *asteya/*non-stealing, *brahmacharya/*moderation *and aparigraha/*non-covetousness. Niyamas refer to five personal practices: *saucha/*purity, *santosha/*contentment, *tapas/*austerity or discipline, *swadhyaya/*self-study or -analysis, *iswara-pranidhana/*surrender to the Divine (see also Bhavanani, 2017).

4 Life Mastery is one of the concepts emphasized in the renewed Norwegian school curriculum (see https://www.udir.no/laring-og-trivsel/lareplanverket/fagfornyelsen/nye-lareplaner-i-skolen/). Life skills are more concrete and general competences one needs in life, promoted by the World Health Organization—WHO (see https://www.skillsyouneed.com/general/life-skills.html).

5
https://worldhappiness.report/ed/2017/

6 The World Happiness Report is an annual report comparing the general happiness in all countries, based on certain agreed-upon parameters. The first World Happiness Report was published in 2012, to support the UN High Level Meeting on global happiness and well-being.

7 See also the Norwegian Public Health Report 2018 (see https://www.fhi.no/nettpub/hin/grupper/psykisk-helse-hos-barn-og-unge/).

8 As much as 4% of Norwegian teen boys received medication for ADHD, while 2% of girls received such medication (https://www.fhi.no/nettpub/hin/grupper/psykisk-helse-hos-barn-og-unge/).

9 Norway has traditionally consisted of a majority population of ethnic Norwegians and a Sami minority population. However, for the last four to five decades the immigrant population has increased rapidly(ssb.no/befolkning/artikler-og-publikasjoner/dette-er-norge-2020).

10 The national partners included both universities, schools, and youth organizations. The Hippocampus project lasted for 26 months and was funded by the Erasmus + program of the EU (see Hippocampusproject.eu).

11 In addition to Yama and Niyama, these limbs include Asana (steady posture), Pranayama (energy/breath control), Pratyahara (sensory withdrawal), Dharana (single minded concentration), Dhyana (meditative state), and Samadhi (state of complete oneness) (see Bhavanani, 2017).

12 see Teenyoga.com

13 The questionnaire was based on the Warwick Edinburgh Mental Well-being Scale (WEMWBS), as well as the Perceived Stress Scale (PSS) and sleep items based on the Patient-Reported Outcomes Measurement Information System (PROMIS)—Sleep Disturbance—Short form scale. The questionnaire was mainly administered as an online survey. In a few cases the questionnaire was also available in paper versions. The statistical analysis was performed by Anju Keetharoth, using STATA, and carrying out paired *T*-test and linear regression analysis. In Norway, Martin Inge Standal validated the statistical data-analysis, focusing on the Norwegian part.

14 The initial coding scheme was produced by Nick Kearny, then discussed jointly in Skype meetings.

15 The thematic analysis of the qualitative interview data was mainly performed manually by Solbjørg Skjelstad, informed by discussions with Ingunn Hagen and Usha S. Nayar. This analysis was validated by Martin Inge Standal, who performed a thematic analysis of the same data using NVivo12.

16 The names of the interviewees are fictional.

17 All of he identified themes include (1) *Emotional management* (e.g., emotional processing and emotional regulation), (2) *Improved sleep regulation* (e.g., sleeping habits, sleep during relaxation exercise, recognize need for rest, improved sleep quality) (3) *Stress regulation and coping* (with life situation), (4) *Enhanced attention, clarity and focus*, (5) *Improvement in self-image* (self-acceptance, self-appreciation, self-confidence), (6) *Improved body-mind awareness* (connectedness with yoga), (7) *Belonging* (More psychological sense of community, affiliation, openness in yoga class, secure attachment).

18 Metta refers to loving kindness meditation, which was part of the yoga programme given to the students.

19 We find it fitting that our research, being part of the Hippocampus project, paid attention to emotions. The Hippocampus (the small part of the brain resembling the Sea Horse, labeled Hippocampus in Greek and Latin) is after all part of the Limbic system, the part of the brain responsible for emotions.

20
https://hippocampusproject.eu/programme/

21 Contentment or *Santosha* is one of the five Niyamas or personal practices/evolutionary observances.

## Appendix | The Hippocampus project, Interview Guide: Norwegian Student Interviews

1. How did you experience the yoga program?/What do you think about the yoga program?

-Attention to yoga as a whole and specific exercises/8 limbs, sequence exercises.

2. How has yoga affected your life? Have you noticed any changes in your life since you started yoga?

- What about family, friends, hobbies,

- School life: is there anything different about being at school? Has something changed in how to learn new things?

- How does yoga affect your emotions?

- Is something different when it comes to what you think about yourself?/What feelings and thoughts you have about yourself?/How do you feel about yourself?

- Are there any changes in physical health or sleep?

- Is there anything else you know, or think of that has changed?

3. Can you describe any changes you have noticed about other students who have been taught yoga at school?

- What about the other students you do yoga together with in yoga class? Have you noticed anything that has been changed/different about them?

- Can you describe something that is different about them after they started with yoga?

4. If you could decide, what would you change about the course you have attended?

- The positions, relaxation or breathing?

- What are your favorites?/What do you like best?

- Is there anything you learned in the yoga course that you want to take with you further in life?

5. Would you recommend other people to do yoga?/Recommend yoga to others?

- What would you say about yoga if you recommended it to someone? In what way would you recommend others to do yoga?

- Do you want to continue with yoga yourself? How?

- What experiences have you had with the YuvaYoga App?

6. What other experiences related to yoga would you like to share with us?

- Is there anything else we have not asked about that you would want to say?
